# Sex-specific adipose tissue’s dynamic role in metabolic and inflammatory response following peripheral nerve injury

**DOI:** 10.1016/j.isci.2023.107914

**Published:** 2023-09-15

**Authors:** Valentina Vacca, Claudia Rossi, Luisa Pieroni, Federica De Angelis, Giacomo Giacovazzo, Ilaria Cicalini, Domenico Ciavardelli, Flaminia Pavone, Roberto Coccurello, Sara Marinelli

**Affiliations:** 1 National Council of Research - Institute of Biochemistry and Cell Biology, Monterotondo (RM), Italy; 2 Department of Innovative Technologies in Medicine and Dentistry, "G. d'Annunzio" University of Chieti-Pescara, Chieti, Italy; 3 Center for Advanced Studies and Technology (CAST), "G. D'Annunzio" University of Chieti-Pescara, Chieti, Italy; 4 Departmental Faculty of Medicine, UniCamillus - Saint Camillus International University of Health Sciences, 00131 Rome, Italy; 5 European Center for Brain Research/Santa Lucia Foundation IRCCS, 00143 Rome, Italy; 6 Università degli studi di Teramo (UniTE) - Facoltà di Medicina Veterinaria, 64100 Teramo, Italy; 7 School of Medicine, University Kore of Enna, Enna, Italy; 8 Institute for Complex Systems (ISC), National Council of Research (CNR), 00185 Rome, Italy

**Keywords:** Natural sciences, Biological sciences, Physiology, Molecular biology, Neuroscience, Systems biology, Proteomics, Metabolomics

## Abstract

Epidemiological data and research highlight increased neuropathy and chronic pain prevalence among females, spanning metabolic and normometabolic contexts, including murine models. Prior findings demonstrated diverse immune and neuroimmune responses between genders in neuropathic pain (NeP), alongside distinct protein expression in sciatic nerves. This study unveils adipose tissue’s (AT) role in sex-specific NeP responses after peripheral nerve injury. Metabolic assessments, metabolomics, energy expenditure evaluations, AT proteomic analyses, and adipokine mobilization depict distinct AT reactions to nerve damage. Females exhibit altered lipolysis, fatty acid oxidation, heightened energy expenditure, and augmented steroids secretion affecting glucose and insulin metabolism. Conversely, male neuropathy prompts glycolysis, reduced energy expenditure, and lowered unsaturated fatty acid levels. Males’ AT promotes regenerative molecules, oxidative stress defense, and stimulates peroxisome proliferator-activated receptors (PPAR-γ) and adiponectin. This study underscores AT’s pivotal role in regulating gender-specific inflammatory and metabolic responses to nerve injuries, shedding light on female NeP susceptibility determinants.

## Highlights

• Female sex is linked to higher neuropathic pain incidence
• Neuropathy prompts sex-related metabolic rates
• Differential fatty acid, acylcarnitine, energy responses by sex pre- and post-injury
• Adipose tissue’s active role in sex-specific nerve injury response

## Graphical abstract

**Figure:**
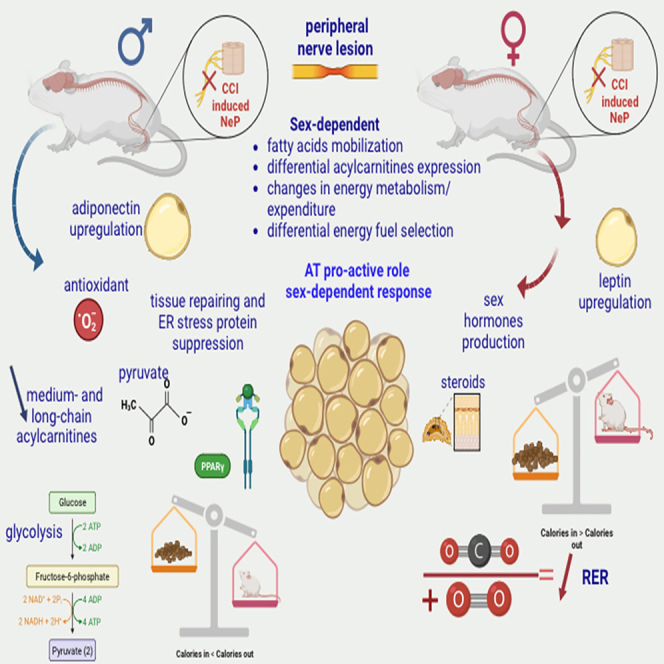

## Introduction

Recently,^1^^,^^2^^,^^3^ the gender bias has been the focus^4^ of intense investigation, especially in the pain field,^5^^,^^6^^,^^7^ both because of the growing number of gender-associated diseases and because sexual dimorphism strongly affects pharmacodynamics and pharmacokinetics. It has been established that females are more pain-sensitive and susceptible to neuropathic pain (NeP) but current knowledge is still far from fully explaining the pain gap.^8^

Our previous paper^5^ established that female mice, even if they had a better response to mechanical stimuli after peripheral nerve injury in the first 15 days from the nerve damage, maintained allodynia without a spontaneous and complete recovery still 121 days after injury (thus differing from males which recover in about 60 days), together with an early decrease of macrophages activation, myelin and spinal cord degeneration and delayed astrocytes and microglia activation.

Peripheral nerve injury induces increased sensitivity to pain and involves primary nociceptors, peripheral nerves, dorsal root ganglia, neurons and glia in spinal cord and brain. In male mice, pain response depends mainly on microglia; in fact, we have shown that in response to peripheral nerve lesion microglia is rapidly activated in the dorsal and ventral horns of the spinal cord seven days after damage and inactivated during the recovery period. On the other hands, in females, microglia appear non-reactive in the first days while it results in strong activation 4 months after the lesion. In female mice the immune system plays a fundamental role in the pain response; in particular, we showed a great involvement of T-cells in peripheral inflammatory response.^6^

This body of evidence strongly suggests that “additional” factors can be involved in the differences observed between male and female mice in the response to NeP. In fact, while neuroimmune and immune systems appear particularly involved in the early stages of neuropathy in males, but not in females, this different activation could be ascribed to the effects of sex-hormones. It is widely demonstrated that testosterone has an immunosuppressive effect while estrogen enhances the immune system response.^9^ In fact, a treatment with exogenous estrogens, during the first days from the lesion, prevents neuropathy and chronic pain in females, reactivating microglia and astrocytes in the first days as well as inducing anti-allodynic effects also in male mice.^10^

Sex-steroids influence and regulate, with a complex crosstalk, multiple systems and processes including immune system and metabolism with a relationship of interdependence. However, it is still unclear the underlying mechanism(s) through which they interfere in the elicitation of a different dimorphic pattern of NeP, including both neuropathy onset and development.

Of particular interest in this context, is our previous proteomics investigation^5^ which revealed the existence of a sex dimorphism in the metabolism of sciatic nerve: females showed lower basal levels of several enzymes involved in glucose metabolism than males, but higher levels of aldose reductase (AR). AR is the first enzyme in the polyol pathway that is also localized in Schwann cells (SCs), whose hyperactivity has been associated to diabetic neuropathy.^11^ Glucose enters in the polyol pathway through AR, and the subsequent accumulation of sorbitol can affect nerve functions. The presence of AR activation in hyperglycemia has a fundamental role in generating a negative loop between oxidative stress, apoptosis and the onset and progression of neuropathy. Of importance, mature SCs can release growth factors (including insulin-like growth factors) to block apoptosis and to stimulate axonal regrowth.^12^^,^^13^ Overall, SCs are highly responsive to metabolic signals changing and deranging their functions since they are equipped with different metabolic sensors and receptors (such as 5′AMP-activated protein kinase - Ampk-, mammalian target of rapamycin - mTOR-, insulin and glucose receptors^14^); thus, changes in glycemia levels can induce apoptosis, de-myelination as well as SCs autophagy.^15^

## Results

### Metabolic changes induced by peripheral nerve lesion

**Figure 1:**
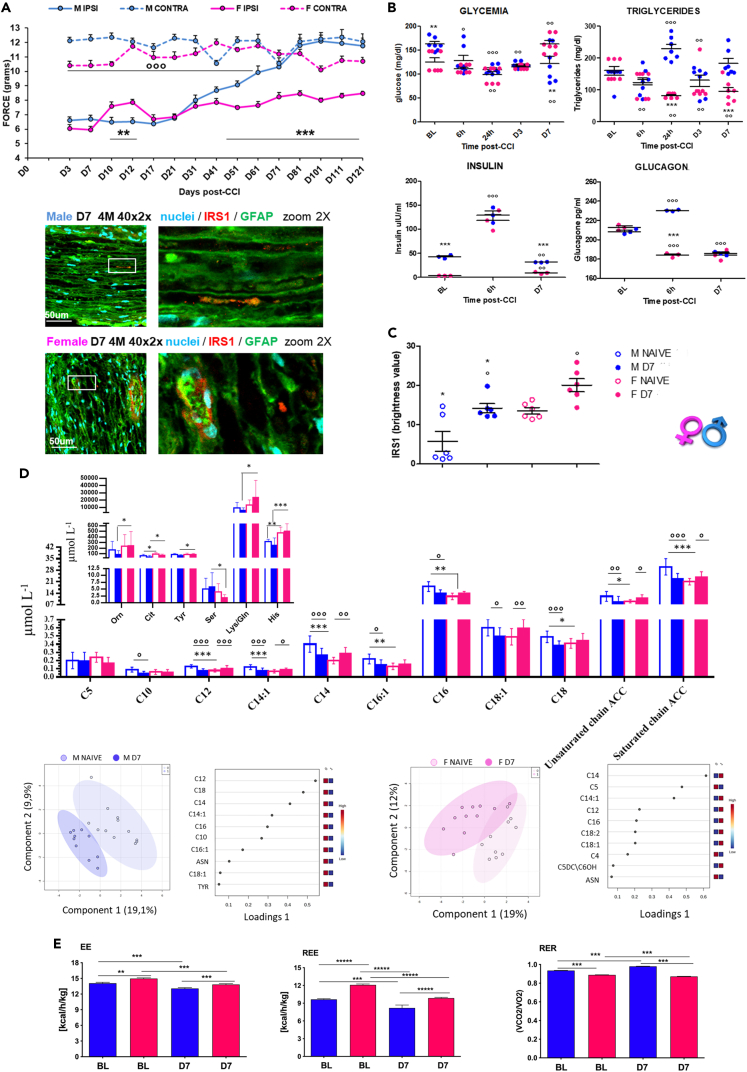
Sex-differences in metabolic response to peripheral nerve injury (A) Sex-related differences in the development of mechanical allodynia induced by Chronic Constriction Injury (CCI): time course of withdrawal thresholds (expressed as applied force in grams) of hindpaws ipsi- and contralateral to the injury in female (F) and male (M) mice. two-way ANOVA for repeated measures revealed significant main effects for sex (F_1,21_ = 62.170; p < 0.0001), time (F_14,294_ = 55.225; p < 0.0001) and sex × time interaction (F_14,294_ = 18.220; p < 0.0001); N = 10/group. (∗)p < 0.05; (∗∗)p < 0.01; (∗∗∗)p < 0.001 between IPSI male and female mice. (^○^) p < 0.05; (^○○○^) p < 0.001 between IPSI and contra in both male and female mice. (B) Nerve injury-induced sex-dependent metabolic rate. Glucose and triglycerides plasma levels at BL and at different time points from injury (6h, 24h, D3, D7) in male and female mice. ANOVA for repeated measures revealed – for glucose significant effects for time (F_4,12_ = 10,29; p < 0.0001) and sex × time interaction (F_4,48_ = 6,99; p = 0.0002); (∗) p < 0.05 and (∗∗) p < 0.01 vs. female; (^○^) p < 0.05, (^○○^) p < 0.01 and (^○○○^) p < 0.001 vs. BL; - for triglycerides: significant main effects for sex (F_1,12_ = 40,46 p < 0,0001) for time (F_4,12_ = 2,73; p = 0.039) and for sex × time interaction (F_4,48_ = 10.38; p < 0.0001); (∗) p < 0.05 and (∗∗∗) p < 0.001 vs. female; (^○○^) p < 0.01; (^○○○^) p < 0.001 vs. BL. (N = 7/group; *in vivo* measurements of glycemia and triglycerides by mean of multiparameter diagnostic device). Glucagon and insulin levels at BL and at different time points from injury (6h, D7) in male and female mice. Kruskal-Wallis – for glucagon H_5_ = 14,567 p = 0.0124; (∗∗∗) p < 0.001 vs. female; (^○○^) p < 0.01, (^○○○^) p < 0.001 vs. BL; - for insulin H_5_ = 16,15 p = 0.0064 (∗∗∗) p < 0.001 vs. female; (^○○^) p < 0.001; (^○○○^) p < 0.001 vs. BL. (N = 3/group/time-point; *ex vivo* measurements by means of ELISA; results classified as BL are derived from naive animals). (C) Confocal analysis of high magnification (40×2x) IF images from sciatic nerves sections co-stained with GFAP (green, Schwann cells marker) and IRS1 (red, Insulin receptor substrate 1) seven days (D7) after injury in male and female mice. Scale bar: 50 μm. Graphs show expression of IRS1 in male and female BL and CCI (D7) mice by using the RGB method that converts pixels in brightness values. ANOVA F_3,20_ = 12,38 p < 0.0001; (∗) p < 0.05, (∗∗) p < 0.01 vs. female; (^○^) p < 0.05; (^○○^) p < 0.01 vs. BL (N = 6/group/time-point). (D) s-PLDA and Loading plot based on whole blood amino acid (AA) and acylcarnitine (AC) profiling of female and male mince before (NAIVE) and after (D7) injury. Histograms show levels of AAs and ACCs expressed as μmol/L N = 10–12/group (∗)p < 0.05; (∗∗)p < 0.01; (∗∗∗)p < 0.001 vs. female; (^○^)p < 0,05; (^○○^)p < 0,01; (^○○○^) p < 0.001 vs. BL. (E) Indirect calorimetry analysis. The left panel shows energy expenditure (EE) kcal/h/kg in male and female mice at BL and D7 after CCI. One-way ANOVA significant main effect for sex/condition (F_3,504_ = 25.36; p < 0.0001) and Tukey-Kramer test post-hoc analysis. The middle panel shows resting energy expenditure (REE) kcal/h/kg at BL and D7 after CCI. One-way ANOVA significant main effect for sex/condition (F_3,238_ = 46.62; p < 0.0001) and Tukey-Kramer test post-hoc analysis. The right panel shows respiratory exchange ratio (RER) VCO2/VO2 in male and female mice at BL and D7 after CCI. One-way ANOVA significant main effect for sex/condition (F_3,504_ = 882.9; p < 0.0001) and Tukey-Kramer test post-hoc analysis; (∗∗) p < 0.01; (∗∗∗)p < 0.001.

Since SCs are the main players in the first days of Wallerian degeneration (WD) and are highly sensitive to metabolic alterations,^16^ we investigated some metabolic parameters (glycemia, insulin, glucagon and triglycerides serum levels) after peripheral nerve injury in male and female CD1 mice subjected to chronic constriction injury^17^ (CCI), which induces WD and allodynia in the ipsilateral hindpaw (Figures 1A and 1B) (all statistical analyses are reported in figures legend).

We found sex-differences in baseline (BL) condition and time- and sex-dependent changes in metabolic rates after the induction of NeP (*in vivo* time-points: BL, 6h, 24h, day 3 - D3 and D7; *ex vivo*: BL, 6h, D7). Neither body weight nor food intake were affected by neuropathy (Figure S1).

Sex-dependent differences in glycemia and insulin levels were observed in BL condition and after CCI. After CCI, a reduction in glucose levels in all time-points was observed in males, while in females after an initial decrease at 24h a significant enhancement in comparison with BL condition was measured at D7. An early (6h) upregulation of insulin was found in both sexes, restoring later to BL value at D7 in males while maintaining a bit higher level in females. Although no sex differences were observed in BL condition, glucagon and triglycerides (TGs) were modulated by sex and by the induction of CCI. Upon CCI and NeP development, glucagon was significantly decreased in females at 6h and D7, while in males it was first upregulated (6h) and finally downregulated (D7). Likewise, serum TGs changes were sex-dependent and strongly dependent on the effects induced by the CCI: TGs were upregulated at 24h and downregulated at D3 in male mice, while they were always downregulated in females.

Since SCs are susceptible to metabolic changes and equipped with insulin receptors, we analyzed the Insulin Receptor substrate 1 (IRS1) in sciatic nerve^18^ for its role in nerve integrity. Sample confocal images and graph in Figure 1C show a sex-dependent expression of IRS1 receptor in BL condition (Naive sample images in Figure S2) and 7 days after nerve injury: neuropathy produced a different increase of IRS1 expression (D7) in sciatic nerves of male and female mice.

Sex-differences are recognized as a crucial factor in energy metabolism and homeostasis of amino acids and fatty acids.^19^ We previously showed^15^ that nerve injury induced several metabolic alterations in adult male mice, suggesting an increase in fatty acids oxidation to cope with the rise of energy need. Thus, targeted profiling of blood amino acids (AAs) and acylcarnitines (ACCs) was performed (Tables S1–S3) to highlight sex differences in the metabolic response to nerve insult. Blood AAs and ACCs profiling were analyzed in male and female mice, showing a good ability to cluster mice before and after injury (Figure 1D).

In BL condition, male and female mice differ for the levels of several medium chain ACCs as well as for unsaturated and saturated medium and long chain ACCs (C12, C14:1, C14, C16:1, C16, C18). In comparison with BL, seven days post-CCI female mice show higher blood level of ACCs (C12, C14:1 C14 and C18:1) while male mice show lower levels of saturated and unsaturated medium and long chain ACCs (C10, C12, C14:1, C14, C16:1, C16, C18:1, C18). After injury, females show lower levels of Serine (Ser), and a trend toward lower levels for C5, a trend toward higher levels for C12, and higher levels of Ornithine (Orn), Citrulline (Cit), Tyrosine (Tyr), Lysine/Glycine (Lys/Gln), Histine (His) in comparison to CCI male mice.

Fatty acids and ACCs mobilization mirror the energy fuel required and, to better understand whole-body energy metabolism and sex-dependent differences before and after CCI, we performed an indirect calorimetry (IC) analysis, thus assessing *in vivo* energy expenditure (EE), resting EE (i.e., in lack of motor activity) (REE) and substrate oxidation (respiratory exchange ratio, RER) (Figure 1E).

The measure of EE, after 48-h continuous recording in BL condition, revealed sex-associated differences in energy metabolism. Female mice showed higher levels of both EE and REE at BL while exhibited lower RER.

Moreover, as compared to BL, peripheral nerve injury at D7 induced a decrease of EE and REE in both sexes. However, the reduction of EE and REE was more pronounced in male than in female mice. As for the RER, female mice showed a lower level at BL that was further decreased after CCI. By contrast, the RER level was increased at D7 in male mice.

From these data emerge that EE and REE are decreased by peripheral nerve damage in both sexes but also that whole-body EE and REE resulted always higher in female animals. The main fuel use appears different between males and females, with an increase of mixed protein/fatty acids oxidation in female mice followed by a switch toward a prevalent increase of fatty acids oxidation after injury. Thus, response to nerve damage can change glucose and fat metabolism, and, in females, enhance EE, in which energy supply seems to rely mainly on the increase of protein and lipid oxidation. To exclude other effects affecting energy metabolism, we evaluated the basal body temperature (BT) and adaptive thermoregulation after exposure to cold temperature. As reported (Figure S3), no significant differences were revealed.

### Role of adipose tissue in orchestrating metabolic response to neuropathy

The secretory activity of adipose tissue (AT), and its involvement in the inter-organ communication has been repeatedly recognized in the last years.^20^ AT is an endocrine organ that is able to regulate glucose, lipids and energy homeostasis, through the secretion of signaling mediators such as adipokines (e.g., leptin and adiponectin), metabolites, fatty acids and steroids. The most part of these investigations were focused on the role of AT in metabolic dysfunction such as in insulin resistance (IR), obesity and diabetes; nothing is known about its potential pathophysiological role in the onset and development of NeP in normo-metabolic subjects.

We previously^21^ showed that leptin was strongly upregulated in sciatic nerve from female mice subjected to peripheral nerve injury. AT-released leptin acts as pleiotropic hormone^22^ in energy metabolism, being involved in the regulation of several homeostatic functions.

To understand whether white adipose tissue (WAT) has an active role in the peripheral neurodegeneration, inflammatory processes, and in sex-dependent metabolic alterations, we performed an analysis of WAT proteomics (Proteomics Data are available via ProteomeXchange with identifier PXD030391). Hence, we made a comparative analysis of the differentially expressed proteins (DEPs) in WAT before (BL) and seven days (D7) after sciatic nerve injury by means of LC-MS based shotgun proteomics.^23^^,^^24^ This experiment allowed us the overall quantification of 525 proteins in the four groups of mice (F_BL; F_D7; M_BL; M_D7). From 525 proteins we filtered out 291 DEPs with a maximum fold change (MFC) of the protein expression levels that was set as MFC ≥1.5 in statistically significant observation (ANOVA p value ≤0.05) (Table S3); details on analysis are reported in supplementary material.

**Figure 2:**
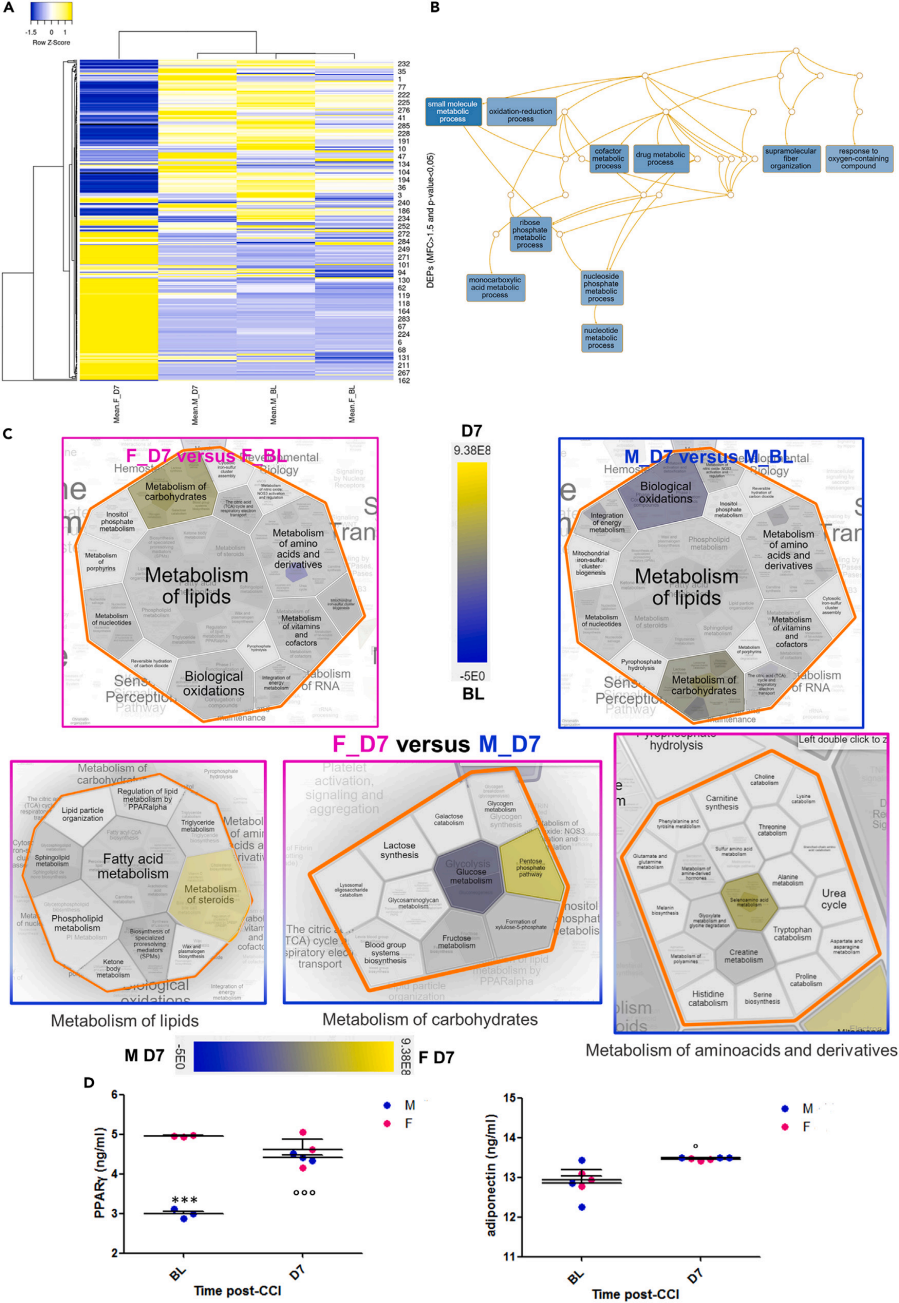
Adipose tissue mobilization in response to neuropathy induction (A) Heatmap representation of the 291 differentially expressed proteins (DEPs) abundance in the four groups. Abundance of a protein is calculated as the mean value of the abundance measured in three replicate runs in LC-MS. (B) DAG (directed acyclic graph) of Gene Ontology enrichment analysis restricted to Biological Process. (C) WAT Proteins whose expression is differentially modulated in F and M upon CCI-D7, enriched Metabolic pathways. Hierarchical Voronoi Plot representing Reactome Pathway Enrichment Analysis. Results of expression enrichment pathway analysis, (scale: blue indicates pathways enriched by proteins with higher means of expression in Baseline -BL condition and yellow at D7) while in the lower three Hierarchical Voronoi Plot blue indicates the higher proteins expression in males after CCI (D7) and yellow in females (D7). (D) PPARγ and adiponectin levels in adipose tissue before (naive mice – BL) and after (D7) peripheral nerve injury in male and female mice. Kruskal-Wallis H_3_ = 8,07 p = 0.044; H_3_ = 8.69 p = 0.033 respectively. (∗) p < 0.05; (∗∗∗) p < 0.0001 vs. female; (^○○^) p < 0.01; (^○○○^) p < 0.001 vs. BL (N = 3/group/time-point).

A heatmap representation (Figure 2A) of the dataset based on protein recurrence illustrates the different expression regulation between females and males, which results of particular relevance at D7 post-injury.

DEPs protein dataset was used to perform gene ontology enrichment analysis with WebGestalt toolkit^25^ (https://www.webgestalt.org/) and pathway enrichment analysis on the Reactome database of pathways and reaction (https://reactome.org/).^26^

Figure S4 shows the enrichment of the complete dataset among the gene ontology categories Biological Process, Cellular Component, and Molecular Function. Interestingly, in the Biological Process bar graph, it is shown that the majority of the protein of this dataset is included in the subcategories biological regulation and metabolic process. More in detail, in Figure 2B, a directed acyclic graph (DAG), shows that different small molecules, metabolic process and oxidative reduction process are enriched in the collected dataset (Table S4).

To further study the involvement of WAT in the metabolic changes after nerve injury, including the GO overrepresentation analysis, we compared males and females at baseline (BL) and seven days after injury (D7), and finally matched males and females at D7. In this last comparison, male mice were considered as the reference group since they showed a complete functional recovery, not observed in female mice (Table S5 resume DEPs relative to each comparison). Volcano scatterplots in Figure S5 allows a quick visual identification of proteins with large fold changes that are also statistically significant in each comparison.

From Reactome Pathway database expression analysis emerges that both in males and females, the proteins upregulated at D7 group are involved in pathways such as glycolysis, gluconeogenesis, creatine metabolism or metabolism of carbohydrates that, being essential metabolic pathways, are able to match the energy requirement (e.g., ATP production and metabolite precursor) for the biosynthesis of small molecules and macromolecules (Figure 2C).

These pathways are also enriched from DEPs in females versus males at D7 after CCI, as well as in other pathways involved in insulin effect on the increase of the small molecules formed during glycolysis and pathways involved in the insulin growth factor (IGF) and Insulin-like growth factor-binding proteins (IGFBPs). A list of the Top 20 ranking pathways enriched for each analysis is shown in Table S6.

Furthermore, if we compare males and females after CCI or females before and after CCI, we observe that WAT of female mice changed after nerve injury with the production of sex hormones and steroids, as revealed by the high presence of proteins such as Steroid receptor RNA activator 1, 20-alpha-hydroxysteroid-dehydrogenase (Akr1c18), estradiol17beta-dehydrogenase5 (Akr1c6) and 3beta-hydroxysteroid-dehydrogenase (hsd3b1 and hsd3b6) (pink letter in Tables S5 and S6), while males utilized antioxidant, tissue repair, protection and suppressors of endoplasmic reticulum (ER) stress proteins (i.e., Alpha-1-Microglobulin/Bikunin Precursor - AMBP-, Serpina 1day, 1a, 1b) (blue letter in Tables S5 and S6).

To validate the idea that WAT is involved in the response to peripheral nerve damage, we measured the expression of Peroxisome proliferator-activated receptor gamma (PPARγ), widely diffused in AT where regulates fatty acid storage, glucose metabolism^27^ and the levels of adiponectin, which is an adipokine involved in the regulation of insulin signaling and inflammatory processes^28^ (Figure 2D). The analysis of adiponectin and PPARγ revealed that adiponectin was upregulated after nerve damage only in males with a parallel increase of PPARγ expression. Of note, adiponectin can influence insulin, glucagon, glucose metabolism, TGs and free-fatty acids concentrations, while PPARγ can change accordingly to medium chain ACCs, being involved in antioxidant activity as well as in peripheral nerve injury and diabetic neuropathy.

## Discussion

In the last years,^29^ AT-derived signaling have been recognized to control and influence other organs and participate to inter-organ communication. AT can be regarded as an endocrine organ regulating glucose and lipid metabolism as well as energy homeostasis throughout the secretion of multiple adipokines such as leptin, fatty acids and steroids.^20^^,^^22^^,^^28^^,^^29^^,^^30^

In spite of this evidence and the literature focused on metabolic disorders in which AT is recognized to play a primary role in the activation of inflammatory events and neurological disorders, there is no knowledge available regarding the potential pro-active role of AT in NeP development.

In seeking for an explanation about why females are more pain sensitive and susceptible to NeP, recent studies^3^^,^^6^^,^^21^ have focused on neuroinflammatory, inflammatory and immune response in female and male mice after peripheral nerve injury, suggesting a specific influence of sex-hormones.

Despite the leading hypothesis for the higher female susceptibility to pain takes mostly into account the different production of endogenous sex hormones,^6^^,^^7^^,^^31^ the literature is still controversial. Indeed, we previously shown^10^ a decrease in both sexes of 17-β-estradiol levels after nerve injury, while estrogen receptors were upregulated after sciatic nerve lesion. Moreover, several experimental investigations,^32^^,^^33^^,^^34^^,^^35^^,^^36^ including ours,^5^^,^^10^ demonstrated that exogenous estrogen prevents neuropathy and pain chronicization and that estrus cycle does not interfere with NeP development. Thus, estrogens appear to protect against chronic neuropathy even though estrogens secretion in females do not seem to confer a higher protection than observed in males, especially considering the major vulnerability to chronic pain observed in the female sex. However, not only ovaries and adrenal glands but also adipocytes are responsible for production of sex hormones in females, and their synthesis from adipocytes can affect whole metabolism, as shown for insulin metabolism.^30^^,^^37^

On one hand, sex-steroids appear as a possible cause of the sexual dimorphic response to NeP development and could account for the different neuroinflammatory, inflammatory and immune response found between males and females. On the other hand, no evidence emerged linking the direct action of these hormones to the mechanisms underlying the neuropathy onset in order to account for the processes responsible of the sex-dependent neuropathic response.

Sex steroids are synthesized from lipids and one source is the AT, which is an endocrine organ having an active role in the control of inflammatory processes and a master regulator of metabolism.

Starting from the idea that neurological damage causes important metabolic changes and a high increase of energy requirements,^15^^,^^38^ we asked if AT could be involved in these mechanisms in a sex-dependent manner.

Here, we provide evidence for sex-associated metabolic alterations in response to NeP. Moreover, we demonstrated that neuropathy in female mice produced changes in lipolysis and fatty acids oxidation (FAO), as well as an enhancement of whole-body energy expenditure and higher secretion of sex hormones from AT, affecting glucose and insulin metabolism. These data could explain the early anti-inflammatory response and the different immunological profile found in females. By contrast, males showed marked alteration of glycolysis and a decrease of systemic energy expenditure and unsaturated fatty-acids levels. In males, the AT response elicited the secretion of factors involved in regenerative process and in oxidative stress, as well as in the stimulation of peroxisome proliferator activated receptors (PPARs) gamma subtype (PPAR-γ) and adiponectin, which are important for pain management and inflammation.^39^^,^^40^

From this analysis emerges that nerve injury elicited in female mice selective changes of the AT sex-hormones (as showed by AT proteomics), revealing a strong up-regulation of enzymes that catalyze for different steroids, together with leptin upregulation as previously demonstrated,^6^ while no changes were detected for adiponectin and PPAR-γ expression.

It is therefore possible that AT-dependent secretion of leptin and AT sex hormones composition in female mice subjected to nerve injury, might explain not only the inhibition of inflammatory process and immunomodulation but also partly account for the metabolic changes triggered by peripheral neuropathy. It should be reminded that sex steroids affect carbohydrates and liver metabolism,^41^ increasing glycemia and insulin release but decreasing muscle sensitivity to insulin.^42^

Being part of the carnitine pool, the ACCs are mitochondria- and peroxisomes-derived intermediate oxidative metabolites consisting in fatty acids (C2-C26) esterified to a carnitine moiety to facilitate their carrying across the mitochondrial membrane.^43^^,^^44^ For this reason, circulating ACCs can be considered indirect markers of mitochondrial function and fatty acid oxidation (FAO) that can mirror FAO defects or metabolic disorders.^45^ Seven days after nerve injury short-chain ACCs, such as C5, were found reduced in female mice, while circulating medium-chain C12 and long-chain C14:1 and C14 C18 were found increased.

In the last years, several studies have helped to disclose additional mechanisms underlying IR in which IR incidence has been linked to the increase of catabolism of branched-chain amino acids (BCAA) and to the accumulation of intermediary metabolites of FAO that may interfere with insulin sensitivity, and in particular with increased plasma levels of C3 and C5 ACCs.^46^^,^^47^ Interestingly, plasma insulin levels were found reduced at D7 in female mice as compared to male mice that underwent to the same experimental procedure of peripheral nerve injury. Moreover, reduced plasma levels of C5 ACCs in female mice might be indicative of energy production relying for the most part on the increase of lipid oxidation, as further corroborated by the decrease at D7 of whole-body RER (VCO2/VO2), thus revealing an increased ratio of fat-to-carbohydrate utilization. The parallel increase of medium-chain C12 and long-chain C14:1 and C14 ACCs may further suggest the presence of incomplete fatty-acid β-oxidation and therefore increase of energy production/expenditure. Within this context, a decrease of circulating TGs was observed at D7 after nerve injury in female mice that may suggest an increase of FAO. As for the increase of whole-body lipid oxidation (i.e., RER reduction), the IC analysis also disclosed an increase of energy expenditure (EE = Kcal/Kg) in female mice as compared to male mice at D7 after nerve lesion.

This complex metabolic mechanism, having in AT a key player, could explain some early events observed in female mice subjected to experimental neuropathy (i.e., macrophages/microglia and myelin proteins strongly influenced by insulin, glucose metabolism and steroids), in which AT-mediated response appear to interfere with the physiological progression of WD and facilitate the development of chronic NeP, thus underlying a sex-dependent impact of AT on metabolic and inflammatory response after nerve injury.

In male mice subjected to peripheral nerve injury, we observed some AT-dependent changes that activate the glycolytic and pyruvate pathway (as shown by proteomics analysis), as well as an upregulation of both PPAR-γ and adiponectin in AT. PPAR-γ activation is reported to normalize insulin level^48^ as well as to exert a direct action on macrophages.^49^ Moreover PPARβ/δ activation results in FAO and fatty acids utilization^50^ since these receptors are involved in energy balance and lipid biosynthesis. Moreover, adiponectin facilitates the oxidation of nonesterified fatty acids, decreasing glucose plasma levels, TGs and concentration of free fatty acids.^51^^,^^52^ Low adiponectin levels can contribute to diabetic neuropathy,^53^ and the lack of adiponectin exacerbates thermal nociception in mice.^54^ Our data show a marked sexual dimorphism in PPAR-γ expression and higher adiponectin activation that might account for the lower allodynic response found in male mice, which exhibited a progressive decrease of sensitivity to mechanical stimulation up to a complete remission from neuropathy.

For both sexes the metabolic changes observed can be, at least in part, explained as consequence of AT mobilization in response to nerve injury. After peripheral nerve lesion, male mice showed a rapid (6h) insulin and glucagon increase, together with the decrease of glycemia levels. Moreover, TGs levels followed a biphasic trend in which the initial increase (24h) was followed by a subsequent decrease (D3).

A rapid increase of insulin may have beneficial effects on macrophages activation through the upregulation of PPAR-γ^49^ that promotes an anti-inflammatory phenotype as well as the stimulation of phagocytic activity taking advantage of low glucose levels.^55^ Finally, insulin exerts a neurotrophic action (through also IRS1) on sciatic nerve stimulating remyelination processes^56^^,^^57^ while glucose levels are essential for the phenotype of SCs.^58^^,^^59^

By contrast with female mice, both medium- and long-chain AACs were found decreased after nerve lesion (D7) in male mice. The differing profile of medium-chain AACs plasma levels is indicative of an opposite use of the main oxidative substrate after nerve lesion among the two sexes. Indeed, while RER was decreased in female mice with regard to baseline and was lower than RER scores showed by male mice after nerve lesion, RER was conversely increased in male mice in comparison to baseline. An increase of RER is indicative of a decreasing rate of lipid oxidation as main fuel and, therefore, indicative of a decreased ratio of fat-to-carbohydrate utilization. An increase of insulin sensitivity has been found associated with a decrease of long-chain AACs after glucose loading, while no decrease was detected in diabetic rats.^60^ Moreover, the proteomic analysis of AT composition discloses the upregulation of glycolysis and pyruvate pathways observed after CCI in male mice. In line with a decrease of medium- and long-chain AACs, an increase of glucose uptake by AT may be viewed as an index of insulin sensitivity.^61^ In other words, an upregulation of the glycolysis pathway in AT appears to be a downstream effect of the insulin-dependent glucose uptake in AT upon glucose transporter 4 (GLUT4) translocation. Both increase of glycolysis and expression of PPAR-γ after nerve injury in male mice should facilitate fat storage in adipocytes and energy sparing as suggested by the decrease of energy expenditure found at D7, which was reduced as compared to both baseline heat (Kcal/Kg) and heat emitted at the same time point by female animals.

Our data strongly suggests that AT can play a pivotal role in inter-organ communication and in the regulation of metabolic response after nerve injury, since peripheral nerves are highly sensitive to glucose, lipids and hormonal changes.

In summary, we demonstrated that peripheral nerve injury can trigger a metabolic stress that induces AT activation which plays a fundamental role in the orchestration of the sex-dependent response to NeP. These data may help to find a missing piece in the puzzle of pain gender gap. A possible new scenario for NeP therapy might be introduced by considering AT and AT-associated targets as new players for the development of gender-specific medicine.

### Limitations of the study

This study aims to uncover novel contributors to the generation of gender-based disparities in neuropathy and the initiation, progression, and recovery of pain. This endeavor seeks to consolidate insights into the mechanisms underpinning the heightened susceptibility and prevalence of neuropathy in females. Our findings unveil AT’s dynamic engagement in a sex-dependent manner within the metabolic response following nerve injury and NeP induction. This active involvement leads to alterations in metabolic rates and the release of both pro- and anti-inflammatory agents that modulate various stages of WD. These revelations introduce a prospective therapeutic landscape for addressing peripheral neural damage, albeit necessitating further exploration to identify potential targets. Given the utilization of an animal model in this research, clinical investigations are imperative to validate these gender-based disparities in AT’s proactive role in neuropathy onset and perpetuation. Moreover, comprehensive exploration is warranted to elucidate the triggers initiating the AT’s responsive behaviors.

## STAR★Methods

### Key resources table

**Table:** 

REAGENT or RESOURCE	SOURCE	IDENTIFIER
**Antibodies**		

Glial Fibrillary Acidic Protein antibody produced in mouse	Sigma-Aldrich	Cat# G6171, RRID:AB_1840893
Insulin receptor substrate 1 antibody produced in rabbit	Abcam	Cat# ab131487, RRID:AB_11156316
Alexa Fluor® 488 AffiniPure Donkey Anti-Mouse IgG (H+L)	Jackson ImmunoResearch	Code: 715-545-150 RRID:AB_2340846
Rhodamine (TRITC) AffiniPure Goat Anti-Rabbit IgG (H+L)	Jackson ImmunoResearch	Code: 111-025-144 RRID: AB_2337932

**Chemicals, peptides, and recombinant proteins**		

bisBenzimide H 33258	Sigma-Aldrich	14530
Formic Acid 99% ULC/MS-CC/SFC	Biosolve	Cat N° 069141
Water ULC/MS-CC/SFC	Biosolve	Cat N° 232141
Acetonitrile ULC/MS-CC/CSF	Biosolve	Cat N° 012141
Sequencing Grade Modified Trypsin	Promega	Cat N° V5111
[Glu1]-Fibrinopeptide B Standard	Waters	SKU: 700004729
MassPREP Enolase Digestion Standard	Waters	SKU: 186002325

**Critical commercial assays**		

ELISA kit Mouse Insulin	RayBio® RayBiotech Inc	P01325
ELISA Glucagon Immunoassay	Quantikine® R&D Systems	DGCG0
ELISA kit Mouse Peroxisome Proliferator Activated Receptor Gamma (PPARg)	MyBiosource Inc.	MBS2087685
ELISA Kit Mouse Adiponectin/Acrp30	Quantikine® R&D Systems	MRP300
NeoBase™ Non-derivatized Assay Solutions	PerkinElmer Inc. Waltham, Massachusetts, United States	Part Number 3040-0010
PerkinElmer 226 Five Spot RUO Card	PerkinElmer Inc. Waltham, Massachusetts, United States	Part Number GR2261002

**Deposited data**		

Raw and analyzed proteomics data	This paper	Data are available via ProteomeXchange Consortium (https://www.proteomexchange.org/) with identifier PXD030391
Raw and analyzed metabolomics data	This paper	Marinelli, sara (2023), “Adipose tissue is a pro-active player in sex-dependent response to neuropathy.”, Mendeley Data, V1, https://doi.org/10.17632/k5vdzdpkvr.1
Raw and analyzed in-vivo, immunohistochemical and ELISA data	This paper	Marinelli, sara (2023), “Adipose tissue is a pro-active player in sex-dependent response to neuropathy.”, Mendeley Data, V1, https://doi.org/10.17632/k5vdzdpkvr.1

**Experimental models: Organisms/strains**		

CD-1® IGS Mouse Crl:CD1(ICR) Outbred	Charles River	RRID:IMSR_CRL:022
Mouse CD1 outbred	EMMA-Infrafrontier (Monterotondo – RM, Italy)	https://www.infrafrontier.eu/emma/

**Software and algorithms**		

LAS X Life Science Microscope Software Platform	Leica Microsystems	https://www.leica-microsystems.com/products/microscope-software/p/leica-las-x-ls/downloads/
ImageJ software	National Institutes of Health - USA	https://imagej.nih.gov/ij/download.html
Statview 5.0 software	SAS Institute Inc.	https://www.macintoshrepository.org/658-statview-5
R Studio	Posit Software	https://posit.co/products/open-source/rstudio/
Porgenesis QI for Proteomics, V4.1	nonlinearDynamics-Water	
Reactome Pathway Database	open-source	www.reactome.org
Panther Classification System	open-source	www.pantherdb.org
MassLynx V4.0 Software	Waters Corp. Milford, Massachusetts, United States	https://www.waters.com/waters/en_US/MassLynx-MS-Software/nav.htm?locale=en_US&cid=513662
GraphPad Software 6.0	Prism - GraphPad	https://graphpad-prism.software.informer.com/6.0/
G∗Power 3.1	Heinrich-Heine-Universität Düsseldorf	https://www.psychologie.hhu.de/arbeitsgruppen/allgemeine-psychologie-und-arbeitspsychologie/gpower
BioRender		BioRender.com

### Resource availability

#### Lead contact

Further information and requests for sources and reagents should be directed to and will be fulfilled by the corresponding author, Sara Marinelli, PhD (sara.marinelli@cnr.it).

#### Materials availability

The study did not generate new unique reagents. Antibodies and biochemical assays used were provided in key resources table, and they are commercially available.

### Experimental model and study participant details

#### Mice models of chronic constriction injury-induced neuropathic pain (NeP)

CD1(ICR)CD1 male and female mice, about 4 months old from Charles River Labs (Como, Italy) or European Mouse Mutant Archive – EMMA Infrafrontier (Monterotondo RM, Italy) were used. Animals were housed in standard transparent plastic cages, in groups of 4, lined with sawdust under a standard 12/12-h light/dark cycle (07:00AM/07:00PM), with food and water available *ad libitum*. Testing was performed blind as for the treatment for treatment group to which each subject belonged. After behavioral testing, the oestrous cycle was analyzed in females by means of vaginal smears. Because we did not observe any difference in the behavioral responses, we included all females in the same experimental group independently from the oestrous cycle. All procedures were in strict accordance with the European and Italian National law (DLGs n.26 of 04/03/2014, application of the European Communities Council Directive 2010/63/UE) on the use of animals for research (Italian Ministry of Health authorization n. 32/2014PR) and with the guidelines of the Committee for Research and Ethical Issues of IASP.^62^

Following the procedure originally proposed by Bennett and Xie^17^ adapted to the mouse, the Chronic Constriction Injury (CCI) model was used as model of NeP. CCI of sciatic nerve was performed under anesthesia with a mixture 1:1 of Rompun (Bayer 20 mg/ml; 0,5 ml/kg) and Zoletil (100 mg/ml; 0,5 ml/kg); the middle third of the right sciatic nerve was exposed through a 1.5 cm longitudinal skin incision. Three ligatures (7-0 chromic gut, Ethicon, Rome, Italy) were tied loosely around the sciatic nerve. The wound was then closed with 4-0 silk suture. In the following, the injured right hindpaw will be named as ipsilateral paw and the uninjured left hindpaw will be named as contralateral paw.

**Figure 3:**
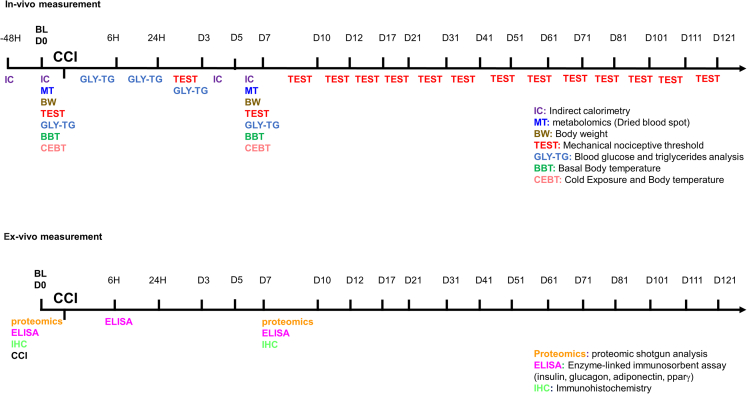
Timeline of experimental procedures On the line, the hour (6h- 24h–48h) or the day of test (D0 to D121) before (baseline - BL) or after CCI are reported. In multicolor acronyms are indicated *in vivo* (top panel) or *ex vivo* (bottom panel) experiments.

Animals were tested (*in vivo* experiments) or sacrificed for tissues collection (ex-vivo experiments) before CCI surgery for obtaining values of reference (baseline). The experimental procedures followed the timeline as reported in Figure 3.

The experimental groups were male and female CD1 mice evaluated before and after (at different time points) the CCI surgery in in-vivo experiments.

A first group of mice were subjected to the following order from the less to the more stressful procedure to avoid experimental interference: allodynia, glycemia and TGs measurements, Body Temperature measurement and cold exposure+Body Temperature measurement.

A second group was designed to the Indirect calorimetry.

For ex-vivo experiments each time-point considered represents a group of male or female CD1 mice since they are sacrificed.

The number of animals (N) of each group is reported in all figure’s legends referring to the specific experiment and statistical analysis.

### Method details

#### Allodynia assessment

Mechanical nociceptive threshold (Dynamic plantar aesthesiometer test). The onset of neuropathy was assessed by measuring the sensitivity of both ipsilateral and contralateral hindpaws to normally non-noxious punctuate mechanical stimuli, at different time intervals from postoperative day 3 (D3) to day 121 (D121). The nerve injury-induced mechanical allodynia was tested by using a Dynamic Plantar Aesthesiometer (Ugo Basile, Model 37400), an apparatus that generates a mechanical force linearly increasing with time. The force is applied to the plantar surface of the mice hindpaw, and the nociceptive threshold is defined as the force, in grams, at which the mouse withdraws its paw. At each day of testing, the mechanical withdrawal response of ipsilateral and contralateral hindpaws was recorded for 3 consecutive trials with at least 10 seconds between each trial. The withdrawal threshold was taken to be the mean of the 3 trials. Behavioral test was performed in male and female.

#### Glycemia and triglycerides measurement

Blood glucose and triglycerides was measured using a Multicare Test Strips apparatus (Biochemical Systems International) by tail clipping in naïve animals (BL) and at different time points after CCI (6h, 24h, D3 and D7) in male and female.

#### Enzyme-linked immunosorbent assay (ELISA) for insulin, glucagon PPARγ and adiponectin

The concentrations of insulin and glucagon were measured in the serum using ELISA kits (RayBio® Mouse Insulin ELISA Kit; RayBiotech Inc., Norcross, GA, USA, and Quantikine® ELISA Glucagon Immunoassay; R&D Systems) as previously described.^63^ Serum samples were harvested at 6h and 7 days after CCI (n=3 per time point) in male and female.

White adipose tissue (WAT) was collected from the subcutaneous abdominal (inguinal) adipose panniculus. PPARγ and adiponectin levels, present in adipose tissue lysates, were measured using ELISA kit (MyBiosource Inc. San Diego USA and Quantikine® ELISA Immunoassay; R&D Systems respectively) in male and female mice (n=3 per time point), before and seven days post CCI. All Incubations and washes were performed following the manufacturer’s instructions. Then a substrate solution was added. The intensity of the colored product was directly proportional to the concentration of insulin and glucagon present in the sera and of PPARγ and adiponectin in adipose tissue and was read at 450 nm (with wavelength correction at 570 nm for insulin, glucagon and adiponectin detection).

#### Immunohistochemical analysis

Sciatic nerve of mice belonging to each experimental group was harvested for IF analysis. Animals were sacrificed with sub-lethal dose of a Rompun (Bayer) and Zoletil (Virbac) mixture and perfused with saline followed by 4% paraformaldehyde in phosphate buffer saline (PBS, pH 7.4). Sciatic nerve was removed and kept in immersion for 48h in 4% paraformaldehyde in phosphate buffer saline (PBS, pH 7.4), after cryoprotection with solution of 30% (w/v) sucrose in PBS and maintained at -80°C. Cryostat microtome sections (20 micron) were taken and mounted directly on glass slides. IF analysis was made before (BL) and seven days after CCI (D7) in both male and female mice adult mice at both times point 4 and 12 months old (4M and 12M). For double IF staining, sections were incubated overnight with: anti-GFAP (glial fibrillary acidic protein, Schwann cell marker) antibody (mouse monoclonal, 1:100, Sigma-Aldrich: G6171); and anti IRS1 (Insulin receptor substrate 1 marker) antibody (rabbit; 1:100, abcam). Both antibodies were diluted in Triton 0,3% (Sigma-Aldrich).

After three washings in PBS, sciatic nerve sections were incubated for 2 h at room temperature with fluorescein-conjugated donkey anti-mouse (ALEXA Fluor 488, 1:100, Jackson ImmunoResearch) and Rhodamine anti-rabbit (1:100, Jackson ImmunoResearch 111025144) secondary antibodies in 0.3% Triton. After two washings in PBS, sections were incubated for 10 minutes with bisBenzimide, DNA-fluorochrome (Hoechst, 1:1000, Sigma-Aldrich) in PBS.

To exclude nonspecific signals of secondary antibodies and to ensure optimal results, both control and treated sections have also been stained with secondary antibody alone (negative controls).

#### Confocal images and analysis

Images of the immunostained sections were obtained by laser scanning confocal microscopy using a TCS SP5 microscope (Leica Microsystem). All analyses were performed in sequential scanning mode to rule out cross-bleeding between channels. High magnification (40X) images of sciatic nerve sections were operated by I.A.S. software (Delta Systems, Italy). Quantification was performed by using the ImageJ software (version 1.41, National Institutes of Health, USA). Fluorescence of IRS1 protein observed was quantified (at least 2 slices each nerve) by converting pixels in brightness values using the RGB (red, green and blue) method that is largely applied to detect, digitalize and analyze microscopic images from biological samples by a confocal microscope.^64^

#### Body temperature

Body temperature (BT) was determined rectally, by means of a digital thermometer with the accuracy of one tenth of centigrade (°C). The measurement was performed at BL condition and after 5 h of 4°C exposure in all experimental groups. The same experimental procedure was performed in the same animals also seven days after injury (D7) in male and female.

#### Energy metabolism

Energy expenditure (EE) or metabolic rate (MR), Oxygen consumption (VO2), and respiratory quotient (RQ) were measured by an indirect calorimeter (IC) system (TSE PhenoMaster/LabMaster System®) with a constant air flow of 0.35 L/min. Mice (N= 9/11 for each group) were adapted for 6 hours to the metabolic chamber, and VO2 was measured every 20 minutes in individual mice, starting at 7:00 PM and ending automatically after 48h (12h dark-light phase comparison). Room temperature was kept constant (22°±1°C). RER= volume of CO2 produced/volume of O2 consumed and is an index of substrate use. EE was calculated as EE = (3.815 + 1.232 x VCO2/VO2) x VO2, as provided by the TSE System. The EE and RER for each of the sample points were evaluated across the 48h of recording. Both EE and RER were also analyzed by considering animals’ resting conditions (values included between 0 and 3 activity counts). Locomotor activity was assessed during the IC by the number of infrared beams broken.

Each cage of the IC system is equipped with the InfraMot® device that uses “passive infrared sensors” to detect and record the motor activity of the mouse by the body-heat image and its spatial displacement across time. Any type of body movement was detected and recorded as activity data.

#### Targeted metabolomics by Flow Injection Analysis–Tandem Mass Spectrometry

Dried blood spot (DBS) samples were obtained collecting whole blood from each mouse on filter paper card. 18 amino acids (AAs), free carnitine (C0), and 30 acylcarnitines (ACCs) in DBS samples were analyzed by Flow Injection Analysis–Tandem Mass Spectrometry (FIA-MS/MS), using the NeoBase Non-derivatized MSMS Kit (PerkinElmer Life and Analytical Sciences, Turku, Finland). Whole blood was collected on filter paper card as dried blood spot (DBS) for the determination of AAs and ACCs by the addiction of isotopically labeled internal standards (ISs) for each analyte prior to the extraction. Filter paper disks of 3.2 mm (3.0–3.2 μL whole blood) were punched out from DBS samples and quality controls (QCs) into a polypropylene microtiter plate to which 100 μL of the extraction solution containing ISs were added. The ISs as well as the extraction solution and the QCs were obtained from the Kit (PerkinElmer Life and Analytical Sciences, Turku, Finland). After being covered, the plate was shaken in a thermo mixer for 50 minutes at 700 rpm and 45°C. 75 μL of the supernatant of each well were then transferred to a new microplate, and the plate was placed in the autosampler for analysis. The low and high QCs in duplicate were run in the same way of the real samples. The analysis of metabolite profile in DBS samples was performed using a Liquid Chromatography Tandem Quadrupole Mass Spectrometry LC/MS/MS system (Alliance HT 2795 HPLC Separation Module coupled to a Quattro Ultima Pt ESI, Waters Corp., Manchester, UK). The instrument operated in positive electrospray ionization, with multiple reaction monitoring (MRM) as acquisition mode, using MassLynx V4.0 Software (Waters Corp.) with auto data processing by NeoLynx (Waters Corp.). Autosampler injections of 30 μL were made into the ion source directly by a narrow peek tube for a total run time of 1.8-minute, injection-to-injection. The mass spectrometer ionization source settings were optimized for maximum ion yields for each analyte. Capillary voltage was 3.25 kV, source temperature was 120°C, desolvation temperature was 350°C and the collision cell gas pressure was 3–3.5 e-3 mbar Argon.^63^^,^^65^^,^^66^^,^^67^^,^^68^^,^^69^

#### Label free differential proteomic shotgun analysis

The analysis was performed on total proteins extracted from adipose tissue obtained from four different groups of animals (n=3 mice/poolull x 3 replications): male and female mice, BL or D7.

White adipose tissue (WAT) was collected from the subcutaneous abdominal (inguinal) adipose panniculus.

Proteins concentration was determined and 50 μg of total protein were transferred on a Microcon-10 Centrifugal filter with 10kDa cut off (Millipore) to perform trypsin digestion according to the filter-aided sample preparation (FASP) protocol, that combines both protein purification and digestion, as previously described.^70^ Upon filter aided buffer exchange to Urea Buffer (UB: 8M Urea, 100mM Tris-HCl in water, pH 8.5) denatured proteins were reduced in 8mM DTT in UB (15 min at 56°C) and alkylated in 0.05M IAA in UB (20min at RT). Prior to proteolytic digestion with trypsin, at an enzyme:protein ratio of 1:50 (w/w), buffer was exchanged to 0.05M ammonium bicarbonate (AmBic) solution in water and carried out at 37°C, 16-18 hours. Trypsin digestion was blocked by adding Formic Acid (FA) to a final concentration of 0.2% (v/v) and the peptides were recovered from the filter in 0.05M AmBic, concentrated in a speedvac and stored at -80°C until use.

Upon digestion, peptides have been resuspended in 0.1% Formic Acid (FA), 0.300 μg of each sample was spiked by 300fmol of MassPREP Enolase Digestion Standard (Waters Corp.) Yeast Enolase Digestion Standard (Waters Corp.) as internal internal standard and run in four technical replicates. The separation of tryptic peptides was performed on an ACQUITY MClass System (Waters Corp.) by loading 3 uL of each digested samples onto a Symmetry C18 5 μm, 180 μm × 20 mm precolumn (Waters Corp.) subsequently separated by a 90 min reversed phase gradient at 300 nL/min (linear gradient, 2–40% ACN over 75 min) using a HSS T3 C18 1.8 μm, 75 μm × 150 mm nanoscale LC column (Waters Corp.) maintained at 40°C. Gradient was obtained by using as mobile phases the following solution: A = 0.1% formic acid (FA) in water B = 0.1% formic acid (FA) in acetonitrile (ACN). Separated peptides have been analysed by High Definition Synapt G2-Si Mass spectrometer (Waters Corp) directly coupled to the chromatographic system. MS signals have been detected in MSE a data-independent acquisition (DIA) protocol workflow.^71^ The following mass spectrometer parameters have been used: positive survey polarity of electrospray source (ES+), acquisition mode mass range 50-2000m/z, capillary source voltage 3.2 kV, source T 80°C, cone voltage 40eV, TOF resolution power 20000, precursor ion charge state 0.2-4, trap collision energy 4eV, transfer collision energy 2eV precursor MS scan time 0.5 sec and fragment MS/MS scan time 1.0 sec. Data were post-acquisition lock mass corrected using the doubly charged monoisotopic ion of [Glu1]-Fibrinopeptide B (Waters), sampled every 30 s.

#### Continuum LC-MS data from three replicate runs for each sample have been processed for qualitative and quantitative analysis using the software Progenesis QI for proteomics v4.1 (NonlinearDynamics-Waters Corp)

The qualitative identification of proteins has been obtained by searching in Mus Musculus database (UniProt.Swiss prot release 2021_04) to which the sequence of Enolase 1 proteins from Saccharomyces Cerevisiae (UniProtKB/Swiss-Prot AC: P00924) was appended. Search parameters were set as: trypsin specified enzyme for digestion performed, automatic tolerance for precursor ions and for product ions, minimum 3 fragment ions matched per peptide, minimum 7 fragment ions matched per protein, minimum 1 peptide matched per protein, 1 missed cleavage, carbamydomethylation of cysteines as fixed modification and oxidation of methionines as variable modifications. False discovery rate (FDR) of the identification algorithm was thresholded as ≤ 1% at protein level. For quantitative analysis, the Absolute Quantitation Using HiN option from Progenesis QI for proteomics software was used using the as absolute calibrant, MassPREP Enolase Digestion Standard previously spiked at the same level in every run and identified, where N=3 indicates the three most abundant peptides measured for each protein and the calibrant.^71^ The LC-MS analysis were performed as previously described.^10^^,^^38^^,^^72^ The mass spectrometry proteomics data have been deposited to the ProteomeXchange Consortium via the PRIDE^73^ partner repository with the dataset identifier PXD030391 and 10.6019/PXD030391.

### Quantification and statistical analysis

All values are expressed as mean ± SEM. The sample size, relatively to *in vivo* experiments, was preventively calculated by means the Power analysis (GPower 3.1). Depending on data, statistical analysis was performed either by unpaired t test, 1-way analysis of variance (ANOVA) or 2-way ANOVA for repeated measures while for small samples (N<5 animals) and groups >3, non-parametric analysis was performed by Kruskall-Wallis. Tukey–Kramer test has been used for post-hoc analysis in multiple comparison or t-Test for single comparison. Differences were considered significant at P < 0.05. For statistics Statview 5.0 and Rstudio were utilized.

The effects of sex and CCI on whole blood AA and ACC profiles were investigated by two-factor ANOVA for repeated measures followed by Fisher post-hoc test. Sex and CCI were the independent factors. When data fail the assumption of homoscedasticity, ANOVA was performed after aligned rank transformation (ART) for nonparametric factorial data analysis.^74^ The aligned rank transform for nonparametric factorial analyses using only ANOVA procedures.^74^ Differences were considered significant at P < 0.05. For statistics, GraphPad Software 6.0, Statview 5.0, Rstudio, and Statistica 6.0 (StatSoft, Tulsa, OK, USA), and Metaboanalyst^75^ were used.

## Data Availability

• Dataset (raw data) referring to this paper are shared in Marinelli, Sara (2023), “Adipose tissue is a pro-active player in sex-dependent response to neuropathy.”, Mendeley Data, V1, https://doi.org/10.17632/k5vdzdpkvr.1. • Proteomic Data are available via ProteomeXchange with identifier PXD030391. • This paper does not report original code. • Any additional information required to reanalyze the data reported in this paper is available from the lead contact upon request.
