# Supplementary material for: Sex-specific adipose tissue’s dynamic role in metabolic and inflammatory response following peripheral nerve injury
Source: iScience. 2023 Sep 15;26(10):107914. doi: 10.1016/j.isci.2023.107914 (PMC10561049; doi:10.1016/j.isci.2023.107914)
Supplement: Document S1. Figures S1–S5 and Table S4 [file mmc1.pdf]

iScience, Volume *26*

Supplemental information

Sex-specific adipose tissue's dynamic role in metabolic and inflammatory response following peripheral nerve injury

Valentina Vacca, Claudia Rossi, Luisa Pieroni, Federica De Angelis, Giacomo Giacovazzo, Ilaria Cicalini, Domenico Ciavardelli, Flaminia Pavone, Roberto Coccurello, and Sara Marinelli

Supplemental information

Sex-Specific Adipose Tissue's Dynamic Role in Metabolic and Inflammatory Response following Peripheral Nerve Injury

Valentina Vacca[1+], Claudia Rossi[2,3+], Luisa Pieroni[4,5+], Federica De Angelis[1], Giacomo Giacovazzo[5], Ilaria Cicalini[2,3], Domenico Ciavardelli[6,3], Flaminia Pavone[1*], Roberto Coccurello[5,7*], Sara Marinelli[1*]

[1] National Council of Research – Institute of Biochemistry and Cell Biology, Monterotondo (RM), Italy

[2] Department of Innovative Technologies in Medicine and Dentistry, "G. d'Annunzio" University of Chieti-Pescara, Chieti, Italy

[3] Center for Advanced Studies and Technology (CAST), "G. d'Annunzio" University of Chieti-Pescara, Chieti, Italy.

[4] Departmental Faculty of Medicine, UniCamillus - Saint Camillus International University of Health Sciences, Rome, Italy

[5] European Center for Brain Research/Santa Lucia Foundation IRCCS, 00143 Rome, Italy.

[6] School of Medicine, University "Kore" of Enna, Enna, Italy.

[7]Institute for Complex Systems (ISC), National Council of Research (CNR), 00185 Rome, Italy.

[+]to be considered co-first authors.

*senior authors

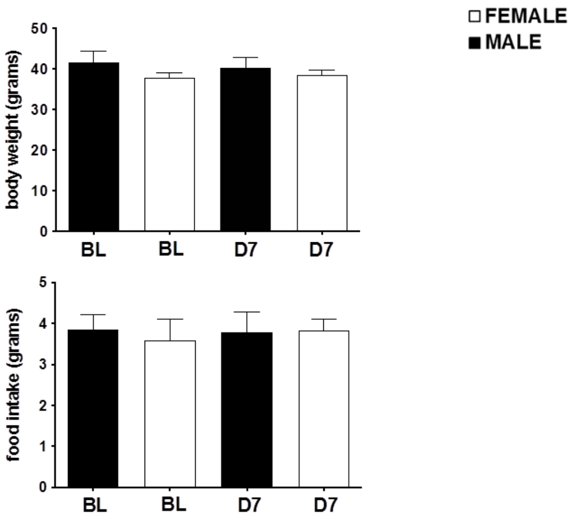

Fig. S1 – Influence of NeP on body weight and food intake. Body weight and food intake recorded before (BL – baseline) and after nerve lesion (seven days –D7 after CCI) in male and female CD1 mice of 4 months old mice. (N=10/group)

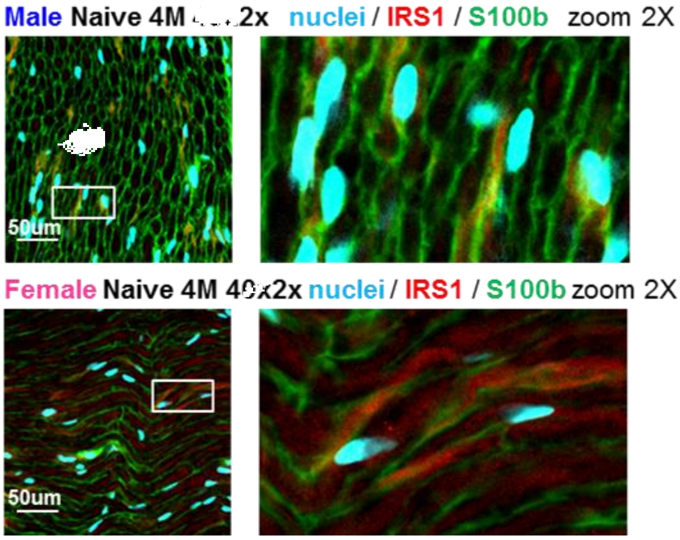

Fig. S2 – Representative confocal images of naïve sciatic nerves. High magnification (40x2x) of sciatic nerves of naïve male and female of 4 months old CD1 mice co-stained for GFAP (green, Schwann cells) and IRS1 (red, insulin receptor substrate 1) markers.

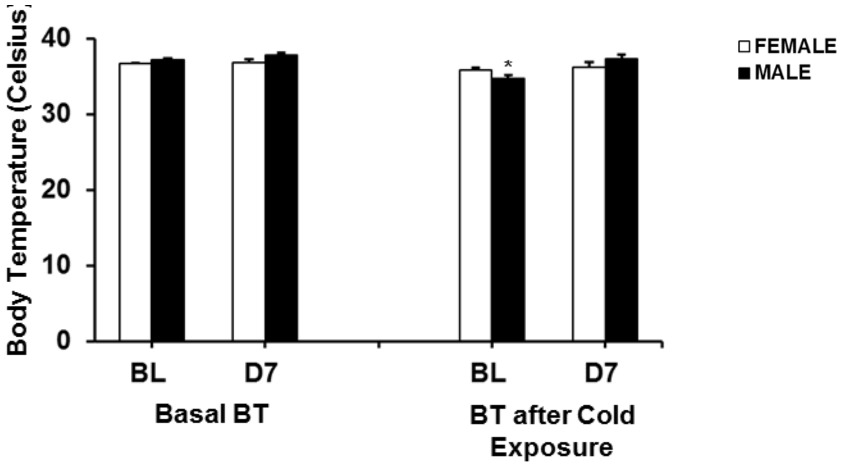

Fig. S3 – Effects of sex and nerve injury on thermoregulation. Basal body temperature (BT) measured before (BL) and after CCI (D7) and after cold exposure in male and female 4 months old mice. No sex-associated differences as well as no appreciable differences were observed with the exception of a significant decrease in male mice in BL condition compared with females after cold exposure (*<0.05 vs female) (n=11/group)

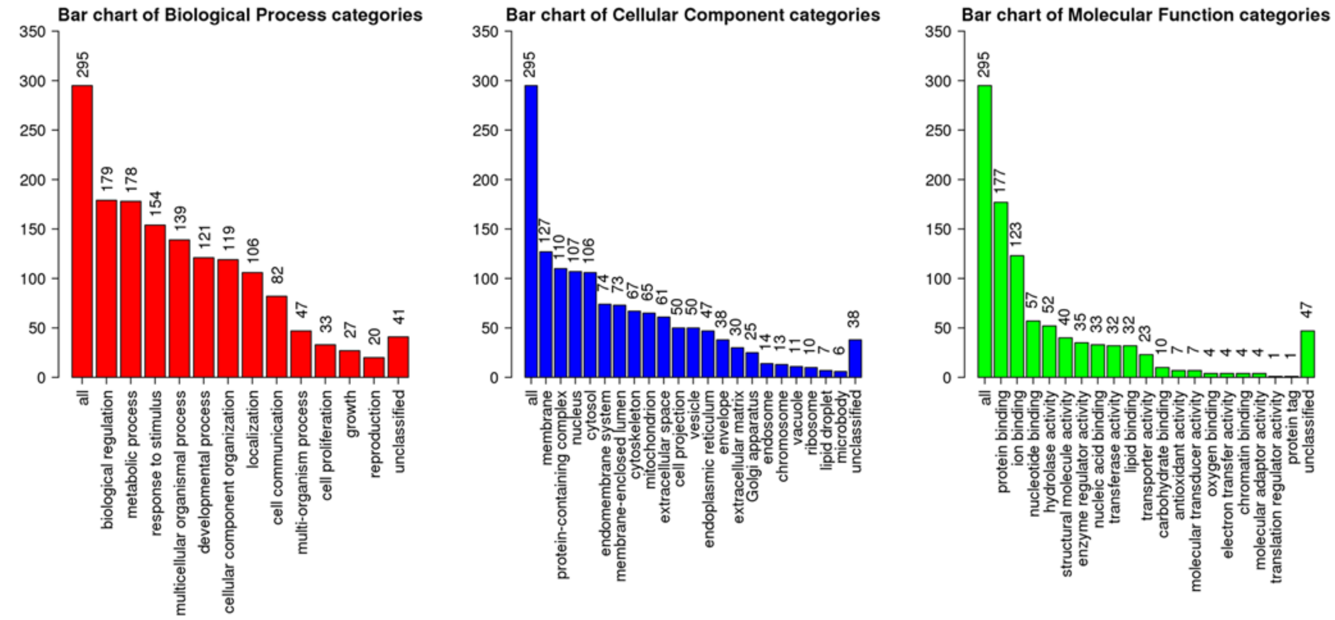

Fig. S4 GO Slim summary for the user 291 quantifiable proteins identified in all groups. Each Biological Process, Cellular Component and Molecular Function category is represented by a red, blue and green bar, respectively. The height of the bar represents the number of IDs in the list and also in the category.

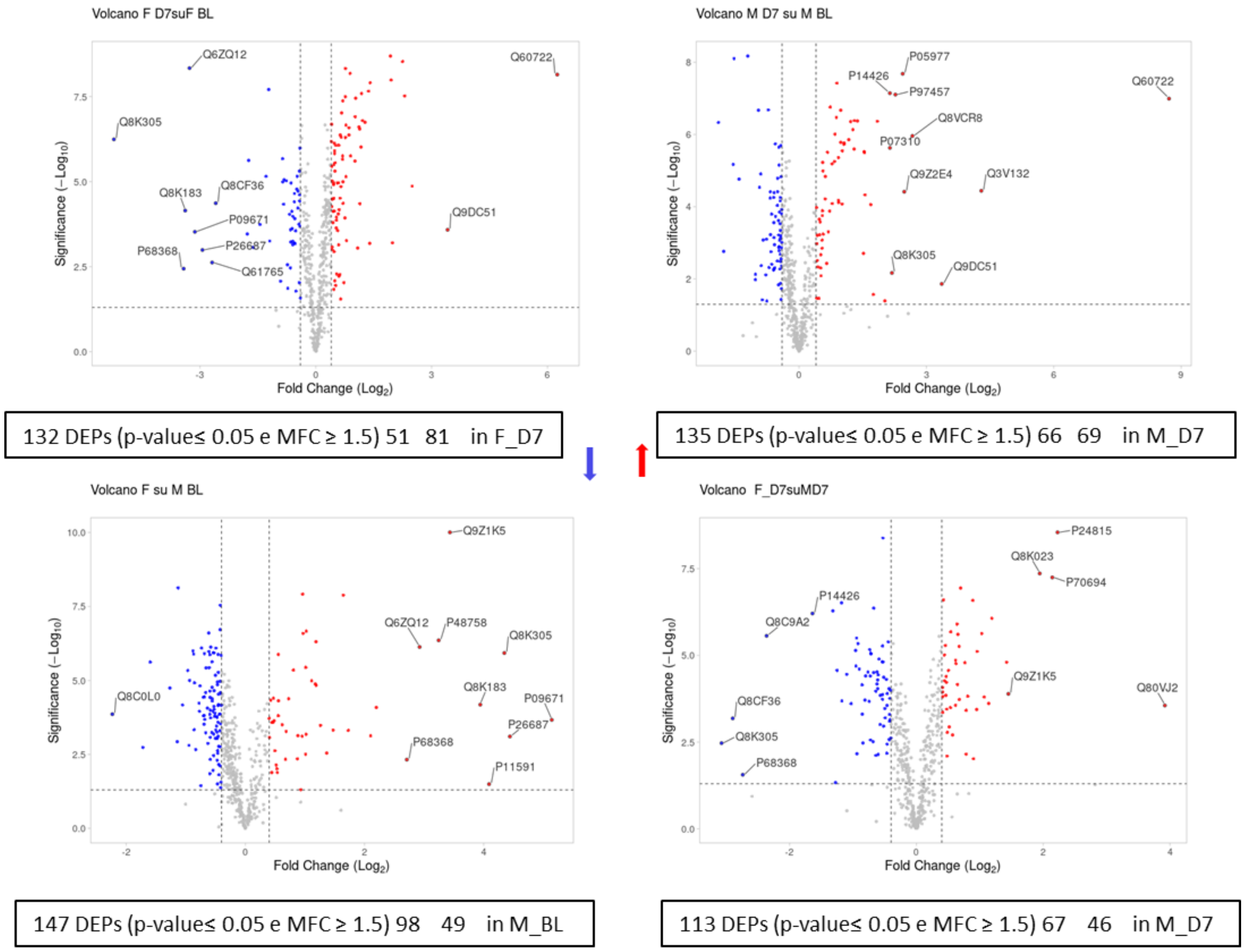

Fig. S5 Volcano Scatter Plots, blue points represent proteins downregulated and red points indicate proteins upregulated. Identification of proteins with large fold changes that are also statistically significant in each comparison

Gene Set	Description	Size	Expect	Ratio	P Value	FDR
GO:0044281	small molecule metabolic process	1736	23.148	31.104	<2.2e-16	<2.2e-16
GO:0051186	cofactor metabolic process	488	65.070	53.788	4,44E-12	2,00E-08
GO:0017144	drug metabolic process	692	92.272	44.434	7,77E-12	2,34E-08
GO:0097435	supramolecular fiber organization	645	86.005	45.346	2,22E-11	5,01E-08
GO:0019693	ribose phosphate metabolic process	443	59.070	54.173	6,88E-11	1,24E-07
GO:0055114	oxidation-reduction process	1026	13.681	35.086	1,87E-10	2,47E-07
GO:0032787	monocarboxylic acid metabolic process	554	73.871	47.380	1,92E-10	2,47E-07
GO:0009117	nucleotide metabolic process	560	74.671	45.533	1,47E-09	1,66E-06
GO:0006753	nucleoside phosphate metabolic process	567	75.604	44.971	2,10E-09	2,11E-06
GO:1901700	response to oxygen-containing compound	1591	21.214	27.811	3,11E-09	2,81E-06

Table S4. Gene Ontology Term
